# Integrated miRNA and mRNA expression profiling of mouse mammary tumor models identifies miRNA signatures associated with mammary tumor lineage

**DOI:** 10.1186/gb-2011-12-8-r77

**Published:** 2011-08-16

**Authors:** Min Zhu, Ming Yi, Chang Hee Kim, Chuxia Deng, Yi Li, Daniel Medina, Robert M Stephens, Jeffrey E Green

**Affiliations:** 1 Transgenic Oncogenesis and Genomics Section, Laboratory of Cell Biology and Genetics, Center for Cancer Research, National Cancer Institute, Building 37, Room 4054, 37 Convent Dr., Bethesda, MD 20892, USA; 2 Advanced Biomedical Computing Center, NCI-FCRDC, Building 430, Room 127, 1050 Boyles Street, Frederick, MD 21702, USA; 3 Laboratory of Molecular Technology, NCI-FCRDC, 915 Toll House Ave, Frederick, MD 21702, USA; 4 Mammalian Genetics Section, National Institute of Diabetes and Digestive and Kidney Diseases, NIH, 10 Center Dr., Bethesda, MD 20892, USA; 5 Lester and Sue Smith Breast Center, Baylor College of Medicine, One Baylor Plaza, Houston, TX 77030, USA; 6 Department of Molecular and Cellular Biology, Baylor College of Medicine, One Baylor Plaza, Houston, TX 77030, USA

## Abstract

**Background:**

MicroRNAs (miRNAs) are small, non-coding, endogenous RNAs involved in regulating gene expression and protein translation. miRNA expression profiling of human breast cancers has identified miRNAs related to the clinical diversity of the disease and potentially provides novel diagnostic and prognostic tools for breast cancer therapy. In order to further understand the associations between oncogenic drivers and miRNA expression in sub-types of breast cancer, we performed miRNA expression profiling on mammary tumors from eight well-characterized genetically engineered mouse (GEM) models of human breast cancer, including MMTV-*H-Ras*, -*Her2*/*neu*, -*c*-*Myc*, -*PymT*, -*Wnt1 *and C3(1)/SV40 *T*/*t*-antigen transgenic mice, *BRCA1^fl/fl^*;*p53*^+/-^;MMTV-*cre *knock-out mice and the *p53^fl/fl^*;MMTV-*cre *transplant model.

**Results:**

miRNA expression patterns classified mouse mammary tumors according to luminal or basal tumor subtypes. Many miRNAs found in luminal tumors are expressed during normal mammary development. miR-135b, miR-505 and miR-155 are expressed in both basal human and mouse mammary tumors and many basal-associated miRNAs have not been previously characterized. miRNAs associated with the initiating oncogenic event driving tumorigenesis were also identified. miR-10b, -148a, -150, -199a and -486 were only expressed in normal mammary epithelium and not tumors, suggesting that they may have tumor suppressor activities. Integrated miRNA and mRNA gene expression analyses greatly improved the identification of miRNA targets from potential targets identified *in silico*.

**Conclusions:**

This is the first large-scale miRNA gene expression study across a variety of relevant GEM models of human breast cancer demonstrating that miRNA expression is highly associated with mammary tumor lineage, differentiation and oncogenic pathways.

## Background

MicroRNAs (miRNAs) are small (19 to 25 nucleotides), non-coding, endogenous RNAs that were first discovered in *Caenorhabditis elegans *during genetic screens for regulators of developmental timing [1-3]. Altered expression of miRNAs has been associated with many human diseases, including cancer [4,5]. Recently, miRNAs have been shown to play important roles in tumorigenesis through their altered regulation of genes involved in cancer development and maintenance. Iorio *et al*. [4] described a breast cancer signature composed of 29 miRNAs that distinguished tumors from normal tissue with an accuracy of 100%. Several miRNAs - miR-10b, miR-373, miR-520c, miR-335 and miR-206 - appear to promote late stages of mammary tumor progression by impacting critical steps in the metastatic cascade such as epithelial-to-mesenchymal transition (EMT), apoptosis, and angiogenesis [6].

In addition to mRNA gene expression profiling, miRNA expression analyses of human breast cancers have further demonstrated another layer of the molecular diversity of this disease and may potentially be a useful diagnostic and prognostic tool for breast cancer therapy and treatment. Blenkiron *et al*. [7] observed that a subset of miRNAs were differentially expressed in the subgroups of mammary tumors originally described by Sorlie *et al*. [8]: luminal A, luminal B, basal-like, HER2+ and normal-like breast tumor subtypes. Moreover, specific miRNAs have been associated with clinicopathological features of breast tumors, such as grade, stage, vascular invasion, estrogen receptor (ER), progesterone receptor, and HER2 status [7,9]. Interestingly, a group of miRNAs, including miR-221/222, miR-206, miR-18a, and miR-22, have been reported to be involved in the regulation of ERα at either the transcriptional or post-transcriptional level [10,11], thereby presenting attractive targets for therapeutic intervention in ERα-negative breast cancer. The molecular distinctions between the various subtypes of breast cancer are critical since they are highly associated with prognosis and response to therapies. Patients with tumors of a basal, hormone receptor- and Her2-negative phenotype generally have a poorer prognosis than patients whose tumors express hormone receptors and are responsive to hormone therapy.

Genetically engineered mouse (GEM) models have been designed to emulate genetic alterations found in human breast cancers. Targeted over-expression of a particular oncogene or knockout of a specific tumor suppressor gene in a well defined genetic background offers particular advantages for studying mammary tumor progression initiated by genetic aberrations relevant to human breast cancer [12]. Moreover, integrated human and mouse gene expression analyses of mammary tumors have revealed that certain mouse tumor models share important similarities to subsets of human breast tumors, including proliferation [12] and tumor subtype signatures [13]. In particular, models with loss of function of *p53, Rb *or *BRCA1 *share molecular features with the human basal-subtype of breast cancer [14].

In this study, we have performed global miRNA expression profiling on eight well-characterized GEM models of human breast cancer (Table 1), including mouse mammary tumor virus (MMTV) long terminal repeat (LTR) promoter driven *H-Ras *[15], *Her2/neu *[16], *c-Myc *[17], polyoma middle T antigen (*PymT*) [18], and *Wnt1 *[19] transgenic mice; C3(1)/simian virus 40 (SV40) *T/t*-antigens (C3(1)/Tag) transgenic mice [20]; *p53^fl/fl ^*;MMTV-*cre *transplant model mice [21]; and *BRCA1^fl/fl^*;*p53*^+/-^;MMTV-*cre *mice [22]. We have identified significant differences in miRNA expression patterns between tumors with luminal or basal-features and for tumors arising from specific initiating oncogenic drivers. We further performed an integrated analysis across all of the mouse mammary tumor samples to identify miRNAs whose expression correlated with the inverse expression of mRNA targets predicted *in silico*. These analyses have identified potential *in vivo *mRNA targets of specific miRNAs in the context of these models of mammary cancer. To our knowledge, this is the first large-scale analysis of miRNA expression in multiple GEM models of mammary cancer and suggests that miRNA expression patterns strongly reflect the lineage subtype of the tumor.

**Table 1:** Summary of mouse mammary tumor models

Model	Number of tumors	Promoter	Strain	Reference
Basal				
C3(1)/SV40 *T/t-antigens*	5	C3(1)	FVB	[20]
*p53^fl/fl ^*;MMTV-*cre *transplant	7	MMTV	Balb/C	[21]
*BRCA1^fl/fl^*;*p53^+/-^*;MMTV-*cre *	5	MMTV	129B6/FVB	[22]
				
Luminal				
MMTV-*H-Ras*	5	MMTV	FVB	[15]
MMTV-*Her2/neu*	5	MMTV	FVB	[16]
MMTV-*c-Myc*	5	MMTV	FVB	[17]
MMTV-*PyMT*	6	MMTV	FVB	[18]
MMTV-*Wnt1*	4	MMTV	FVB	[19]

MMTV: mouse mammary tumor virus promoter, often expressed in virgin mammary gland epithelium, induced with lactation; often expressed at ectopic sites (for example, lymphoid cells, salivary gland, others). C3(1): 5' flanking region of the C3(1) component of the rat prostate steroid binding protein, expressed in mammary ductal cells and at low levels in other tissues. PyMT: polyoma middle T antigen.

## Results

### miRNAs are differentially expressed among GEM mammary tumors

A custom miRNA microarray platform was used to generate miRNA expression profiles of the eight GEM models of human breast cancer, including 42 primary tumors from individual mice and 5 normal mammary glands from 17.5-day-pregnant female mice (Table 1). Since mammary tumors are composed primarily of epithelial cells, we chose to use pregnant mammary glands that are highly enriched for mammary epithelial cells, which are much less represented in virgin mouse mammary glands that contain a very high component of fat cells.

Since the *p53^fl/fl ^*;MMTV-*cre *and *BRCA1^fl/fl^*;*p53*^+/-^;MMTV-*cre *tumors were derived from mice with different strain backgrounds compared to the other models in the FVB/N background (Table 1), we initially determined whether significant differences in miRNA were associated with the various background strains. We identified 22 miRNAs that are differentially expressed in 17.5-day-pregnant mammary glands from FVB, Balb/C and 129B6/FVB mouse strains (Additional file 1). Hierarchical clustering of the expression of these miRNAs across all of the mouse mammary tumor models indicated that the expression levels of the 22 miRNAs in the tumors were not related to the background strain of the mouse (Additional file 2).

**Figure 1:**
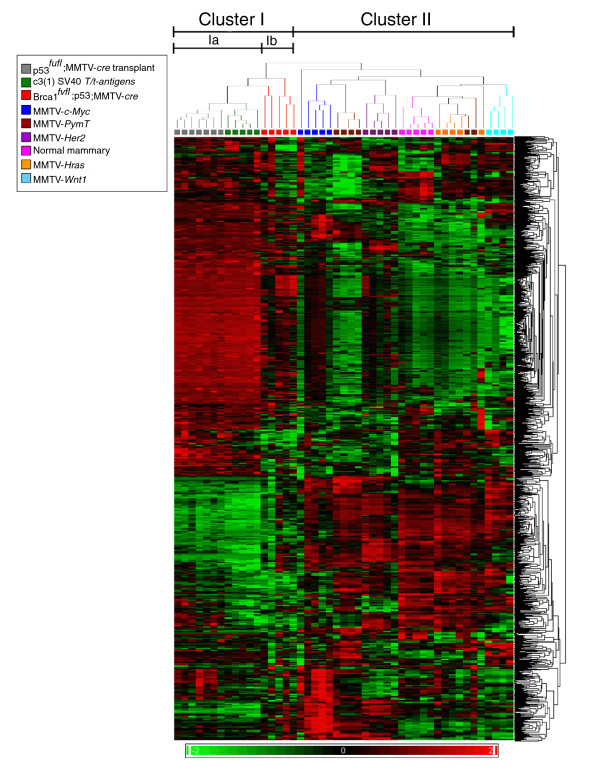
**Unsupervised hierarchical clustering analysis of miRNA gene expression of 41 mammary tumors derived from 8 genetically engineered mouse models and samples of 5 normal mammary glands from 17.5-day-pregnant FVB/N mice**. The heatmap shows the expression of 1,336 mouse miRNAs at the probe level. Heatmap colors represent relative miRNA expression as indicated in the color key.

Unsupervised hierarchical cluster analysis of miRNA gene expression data separated the mouse tumors and normal mammary gland tissues into several clusters that were associated with specific tumor models (Figure 1). Tumors from the *p53^fl/fl^*;MMTV-*cre *transplant, C3(1)/Tag and *BRCA1^fl/fl^*;*p53*^+/-^;MMTV-*cre *models formed one major cluster (cluster I). However, the *p53^fl/fl^*;MMTV-*cre *transplant and C3(1)/Tag models shared the greatest similarities in miRNA expression patterns (cluster Ia); the *BRCA1^fl/fl^*;*p53*^+/-^;MMTV-*cre *model clustered separately (cluster Ib). In contrast, tumors from four of the five MMTV promoter-driven transgenic mice (MMTV-*H-Ras*, MMTV-*PymT*, MMTV-*Her2/neu *and MMTV-*Wnt1*) formed a second major cluster (cluster II). Furthermore, the normal mammary gland tissues from pregnant FVB mice clustered with this group of tumors, suggesting that they may share similar molecular features related to their lineage of origin. Interestingly, a group of human breast tumors has been classified as having a 'normal' subtype with similarities in a gene signature found in normal breast epithelium [7,23]. Within cluster II, MMTV-*Wnt1 *and MMTV-*Her2/neu *each formed separate clusters, whereas normal mammary glands, MMTV-*H-Ras *and 2/6 MMTV-*PymT *tumors clustered together. A subcluster containing four of the five MMTV-*c-Myc *tumors and four of the six MMTV-*PymT *tumors was separated from the remaining three subgroups in cluster II.

These results suggest that the miRNA expression patterns are largely determined by the tumor lineage since the tumors identified in cluster I have been associated with the basal tumor phenotype, whereas the tumors in cluster II have been associated with a phenotype that is clearly distinguished from basal tumors and displays some luminal features (Additional file 3). The inclusion of the normal mammary tissue samples into cluster II further supports the association of this cluster with a luminal phenotype.

### Validation of miRNA expression

A subset of miRNAs that were identified to be differentially expressed among the mouse models by microarray analysis was selected for further validation. Real-time RT-PCR was performed to assess miRNA expression in samples from the various tumor models. Comparison of expression levels between the miRNA microarray data and the PCR results demonstrated a strong correlation between the two platforms for miR-107, -10b, -193, -200b, -494, -505, -7a, and let7f; a modest association for miR-30b, -412; and weak or no association with miR-135b, -155, and -301 (Additional file 4). The poor correlation for some of the miRNAs may be due to differences in sensitivities between the assays, PCR primers, alternative 3' modifications of miRNAs that could significantly influence the sensitivity of the PCR assays or the robustness of the probes on the array.

### miRNA features are associated with mammary tumor differentiation

**Figure 2:**
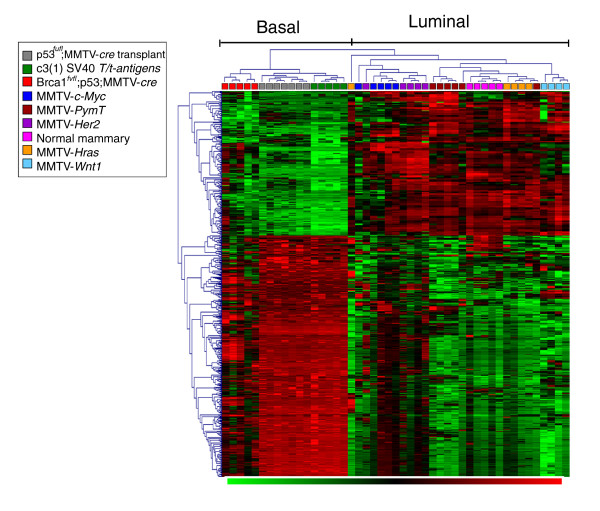
**Hierarchical clustering analysis of basal- and luminal-specific miRNA gene expression among mouse mammary tumor subtypes**. miRNAs that distinguished basal from luminal tumor subtypes were identified and used in this hierarchical clustering of all tumor samples. A color-coded matrix below the dendrogram identifies each sample: red, basal like; green, luminal. The normal mammary samples were then integrated into the heatmap for comparison.

We performed an analysis of miRNA expression data to identify miRNAs that were differentially expressed (*P *≤ 0.01, false discovery rate (FDR) ≤ 0%) between the mouse basal-type (C3(1)/Tag, *p53^fl/fl^*;MMTV-*cre *and *BRCA1^fl/fl^*;*p53*^+/-^;MMTV-*cre*) and luminal-type (MMTV-*H-Ras*, -*Her2/neu*, -*c-Myc*, -*PymT*, and -*Wnt1*, excluding the normal samples) mammary tumors. As depicted in the heatmap in Figure 2, multiple miRNAs are distinctly expressed between the basal-like and luminal-type mammary tumors. The normal mammary gland tissue samples also clustered with the luminal-type mammary tumors.

A total of 122 miRNAs (430 probes) were highly expressed in the basal-like mammary tumors compared to the luminal-type mammary tumors. Seventy-three miRNAs (257 probes) were highly expressed in the luminal-type but not in the basal-like mammary tumors (Additional file 5). Table 2 lists the top 20 miRNAs that were highly expressed in the basal-like and luminal-type mammary tumors.

**Table 2:** Differentially expressed miRNAs among mammary tumor subtypes

Tumor subtype	mmu-miRNA	Fold change	*t*-Test *P*-value	Chromosome
Basal	448	8.0	3.11E-14	X
	201	7.2	7.48E-12	X
	687	7.6	9.75E-12	14
	463	7.2	1.05E-11	X
	713	8.3	4.90E-11	13
	490	9.6	7.22E-11	6
	323	7.2	1.15E-10	12
	137	7.1	1.35E-10	3
	688	8.4	1.17E-09	15
	302b*	9.2	1.58E-09	3
	295	7.7	2.58E-09	7
	592	7.0	4.08E-09	6
	412	9.7	7.13E-08	12
	681	7.2	8.24E-08	12
	464	7.5	1.59E-07	15
	718	8.0	2.05E-07	X
	217	7.6	2.52E-07	11
	465a-5p	8.6	2.82E-07	X
	701	8.7	4.95E-07	5
	693-5p	11.0	1.43E-06	17
				
Luminal	106a	10.8	1.21E-15	X
	106b	12.2	6.70E-15	5
	805	12.4	2.10E-14	MT
	191	9.9	9.20E-12	9
	30c	14.4	4.19E-11	4
	26a	12.6	5.51E-11	9
	19b	15.7	1.11E-10	X
	30b	13.7	2.80E-10	15
	30a	13.4	3.23E-10	1
	30d	10.4	4.64E-10	15
	146b	17.6	7.42E-10	19
	148a	18.6	1.35E-09	6
	193	13.1	2.78E-09	11
	141	20.9	2.95E-09	6
	195	14.8	3.25E-09	11
	26b	15.1	1.16E-07	1
	200a	13.4	6.03E-07	4
	182	13.2	8.78E-06	6
	30e	9.8	1.46E-05	4
	200b	13.8	2.38E-03	4

The highly expressed top 20 miRNAs that are associated with either basal-like or luminal-type mammary tumors. mmu-miR-302b* designated in the miR9.0 release is currently named mmu-miR-302b in the miR17.0 release, but the sequence has not changed [54].

### miRNAs associated with the initiating oncogenic event

Analysis of 334 unique miRNAs (that are each represented by four probes on the microarray chip) demonstrated that despite different genetic drivers used to initiate tumorigenesis, several mouse models share very similar miRNA expression profiles (Figure 1). In order to further define miRNA features that are associated with specific oncogenes or oncogenic pathways, and to determine the fundamental differences in miRNA expression between the normal mammary glands and mammary tumors, we compared the miRNA expression profiles across all of the murine tumor models and normal mammary glands.

**Figure 3:**
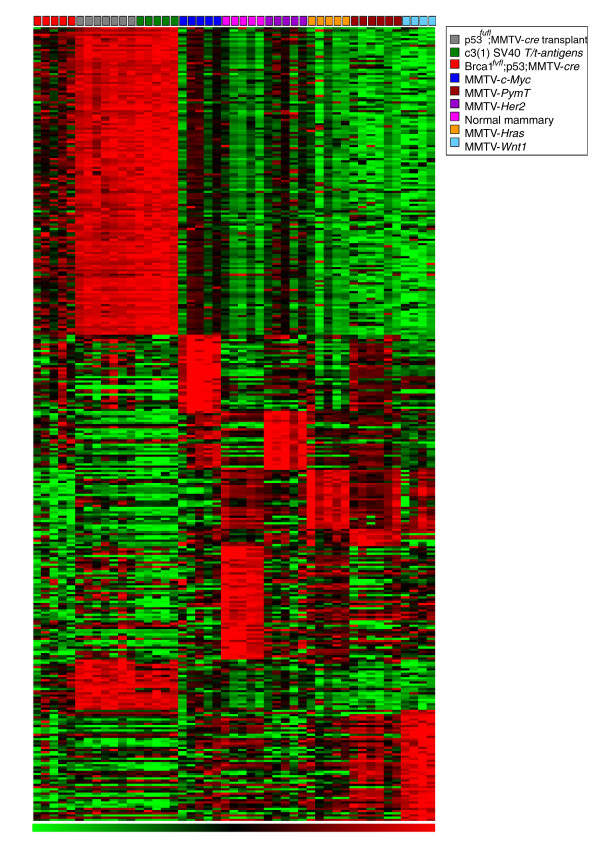
**Heatmap of GEM-specific miRNA expression signatures associated with eight GEM models and normal mammary glands**. In-house z-score-based methods are used with *P*-value < 0.001, FDR by permutation less than or close to 1%, and FDR-BH (false discovery rate-Benjamini and Hochberg) < 5% as described in Materials and methods.

miRNA expression values were converted to z-scores representing the relative expression of each miRNA probe compared to all probes on the array. Model-specific miRNAs were then identified as those most highly expressed among all the samples with a z-score > 0.75, but with no more than two samples from any of the other models having their miRNA expression z-scores higher than the median for the model being evaluated. This algorithm identified clusters of miRNAs that are most highly expressed in one but not all of the other mouse models. The expression of these miRNAs, therefore, may be related to the initiating oncogenic event and may potentially contribute to mammary tumor initiation or progression (Figure 3). A list of model-specific miRNAs is provided in Additional file 6 for all of the GEM models except for *BRCA1^fl/fl^*;*p53*^+/-^;MMTV-*cre*, where no model-specific miRNAs were identified. In addition, we identified a list of miRNAs that are highly expressed only in the normal mammary gland tissues, but not in any of the tumor models (Additional file 6).

### Identification of potential mRNA targets of miRNAs

miRNA recognizes its target mRNA by binding to a 6- to 8-mer 'seed' sequence located on the 3' UTR of the mRNA. Several computational algorithms have been developed in predicting the potential miRNA targets based on the 'seed' sequence, and the three commonly used algorithms are TargetScan, miRanda and PicTar, available through the Sanger miRBase. However, these computer algorithms generate a large portion of false positive miRNA targets. In order to identify potential genes whose mRNAs might be targeted by specific miRNAs, we performed an inverse correlation analysis at the probe level between the expression of a specific miRNA and the expression levels of all the predicted mRNA targets of the miRNA by TargetScan for all of the mammary tumors and normal tissues. This approach identified candidate miRNA target genes that are down-regulated at the transcriptional level and are inversely correlated with the expression of the miRNA in the same corresponding samples. Our analysis yielded putative target mRNAs for a subset of the model-specific miRNAs (Additional file 7), basal-like and luminal-type specific miRNAs (Additional file 8). Only a small subset of the total TargetScan predicted genes were identified as potential miRNA target genes by this analysis. For instance, the expression of only 19 out of 156 TargetScan predicted targets were inversely correlated with the expression of miR-10b, and 9 out of 101 for miR-412 (Table 3). Similarly, as shown in Table 4, only 12 out of 245 predicted targets were found to show an inverse correlation with expression of miR-494.

**Table 3:** Model-specific miRNAs with their potential mRNA targets

GEM model	Model-specific mmu-miRNA	Number of miRNA probes	Number of target genes
MMTV-*c-Myc*	494	4	12
	685	1	8
	699	1	10
			
MMTV-*H-Ras*	182	1	32
	200c	3	28
	30b	4	99
			
MMTV-*Wnt1*	106b	4	26
	130a	1	35
	15a	1	65
	19b	4	19
	22	4	22
	301	1	10
	335	2	4
			
MMTV-*PyMT*	7	2	14
			
MMTV-*Her2/neu*	193	3	8
			
C3(1)/SV40 *T/t-antigens*	412	1	9
			
Normal mammary	10b	3	19
	148a	4	41
	150	1	4
	199a	1	19
	486	4	2

By applying an integrated miRNA-mRNA correlation analysis, mRNA targets are identified for a list of miRNAs associated with normal mammary tissues and individual GEM models. PyMT, polyoma middle T antigen.

**Table 4:** Basal- or luminal-like miRNAs with their potential mRNA targets

Tumor subtype	mmu-miRNA	Number of miRNA probes	Number of target genes
Basal	150	1	4
	219	1	7
	222	1	15
	375	4	3
	412	4	4
	505	2	13
	689	4	2
			
Luminal	100	4	6
	101a	1	60
	101b	2	61
	106a	2	19
	106b	4	28
	130a	1	35
	141	1	15
	148a	4	41
	152	4	40
	15a	1	65
	17-5p	3	29
	182	1	33
	193	3	8
	19b	4	19
	200b	4	23
	200c	4	26
	20a	4	33
	22	4	22
	26a	4	75
	26b	4	59
	27a	1	11
	28	4	7
	30a-5p	4	66
	30b	4	100
	30c	4	111
	30d	4	85
	30e	2	9
	429	3	25
	494	4	12
	685	1	8
	7	1	25
	709	3	14

By applying an integrated miRNA-mRNA correlation analysis, mRNA targets are identified for a list of basal- and luminal-like miRNAs.

Furthermore, we plotted the global distribution of the Pearson correlation coefficients between an miRNA of interest and either all mRNAs that are probed by the Affymetrix array chip (430A 2.0) or only those mRNAs that are predicted targets of the miRNA. For instance, for miRNAs miR-10b, miR-412 and miR-494, the distribution curve of the correlation coefficients for all mRNAs and that for target mRNAs are notably different, with the latter showing a distinct shift that extended towards negative Pearson correlation coefficients (Additional file 9). This pattern is a departure from a normal distribution and indicates that the tissue transcript levels of a subset of mRNAs, which have a predicted miRNA target sequence in the 3' UTR, are reduced by miR-10b, miR-412 and miR-494, respectively. Such a shift in patterns indicates an enrichment for the corresponding negatively correlated mRNAs within the predicted targets (more likely to be the 'true' targets of these miRNAs) of these differentially expressed miRNAs, which were statistically significant as assessed by Fisher's exact test (see Materials and methods).

### Over-expression of candidate miRNA results in inhibition of its target mRNAs in breast cancer cells

In order to determine the functional relationship between an miRNA and its potential targets identified by the miRNA-mRNA inverse correlation analysis, we selected two miRNAs, miRNA-494 and miRNA-412, for further analysis.

Expression of miR-494 was highly associated with the *c-Myc *transgenic model (Table 3), and with the luminal-type mammary tumors (Table 4). Moreover, all four probes on the array for miR-494 have 12 predicted target genes in common. These 12 target genes were analyzed using Ingenuity Pathway Analysis software (Ingenuity Systems, Inc., Redwood City, CA, USA). Core pathway analysis revealed that 4 of these 12 target genes - Bmi1 [24,25], Birc4 [26], Bmpr2 [27] and Ptpn12 [28,29] - have been found to be significantly deregulated in cancer (Additional file 10). Expression of miR-412 (one probe) was shown to be highly associated with C3(1)/Tag tumors and nine potential target genes (Table 3). The expression of four miR-412 probes was also associated with basal-like tumors (Table 4) and four predicted target genes, including *Bmpr1a, Foxo3 *and *Spry4 *(Additional file 8). These genes have been associated with breast cancer tumorigenesis [30-33]. Additionally, *Bmpr1a *is a predicted target for all of the four miR-412 probes.

We transfected two mouse mammary tumor cell lines, M6 and DB7, with lentivirus expressing miR-494 and miR-412, respectively. M6 cells were derived from a primary C3(1)/Tag tumor [34] and express low levels of miR-494, but relatively high levels of miR-412. DB7 cells were derived from a primary MMTV-*PymT *tumor [35] and express low levels of miR-412 but relatively high levels of miR-494. M6 cells stably expressing miR-494 (M6-miR-494) or scrambled miRNA (M6-scramble) and DB7 cells stably expressing miR-412 (DB7-miR-412) or scrambled miRNA (DB7-scramble) were established using puromycin selection and fluorescence activated cell sorting (FACS) sorting for red fluorescence protein (RFP) expression. Increased expression of miR-494 and miR-412 was confirmed in the M6-miR-494 (Additional file 10) and DB7-miR-412 cells compared to control cells expressing scrambled miRNA. No miR-412 was detectable in control DB7 cells by quantitative RT-PCR after 40 cycles whereas miR-412 was detectable in DB7-miR-412 cells at threshold cycle 31. A 1.9-fold increase in miR-494 expression was identified in M6-miR-494 cells compared to control M6 cells (*P *= 0.009; Additional file 11).

**Figure 4:**
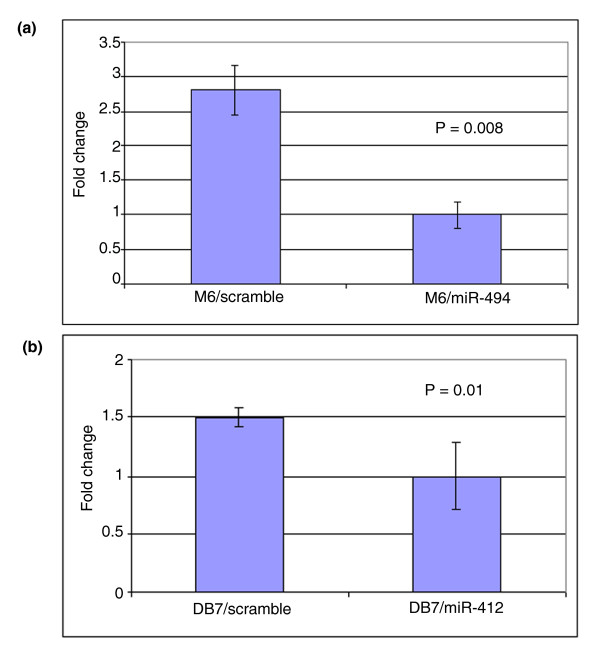
**Over-expression of (a) miR-494 and (b) miR-412 inhibits expression of *Birc4 *and *Bmpr1a*, respectively**. M6 cells and DB-7 cells were transduced with lentivirus expressing miR-494 and miR-412, respectively. Control cells were transduced with lentivirus expressing scrambled miRNA. Following infection, cells were FACS sorted for RFP and RNA was extracted. RT-PCR was then performed to examine the expression of *Birc4 *in M6 cells and *Bmpr1a *in DB-7 cells. The error bar represents the standard deviation.

Quantitative real-time PCR revealed that expression of *Birc4 *was significantly reduced in M6-miR-494 cells but not in control cells (*P *= 0.004; Figure 4a). However, there was no detectable change at the transcript level for *Bmi1 *and *Ptpn12 *in these cells (Additional file 12). Expression of *Bmpr1a *was decreased 1.5-fold in DB7-miR-412 cells compared to that of control cells (*P *= 0.02; Figure 4b). However, increased expression at the transcript level was observed for *Foxo3a *and *Spry4 *in these cells (Additional file 13).

## Discussion

Genome-wide miRNA expression analyses and functional studies have revealed important roles for these small regulatory molecules in breast cancer biology. This study of miRNA expression in relevant GEM models of human breast cancer provides the opportunity to distinguish miRNA expression patterns in a supervised manner according to the known molecular alterations that induce tumor formation and characteristics of the tumor phenotype. The miRNA expression patterns can be further interpreted based upon our previous studies that have delineated gene expression patterns for these same GEM models [13,14]. This is the first large-scale miRNA gene expression study across a variety of GEM models of human breast cancer and strongly suggests that a primary determinant of miRNA expression is the lineage of the tumor (that is, basal versus luminal), supporting the previous report that altered miRNA expression is confined to specific epithelial cell subpopulations in human breast cancer [36].

We chose to analyze these eight GEM mammary tumor models since they have been designed to initiate tumorigenesis through different molecular pathways that are quite relevant to human breast cancer. We identified miRNAs that are associated with specific models or that are commonly deregulated in all of the mammary tumors models. Unlike similar studies involving human patient samples, genomic analyses of GEM models may be performed in defined genetic backgrounds, which greatly reduces variability in expression due to genetic variation as is often the case in human studies. The results of this study have demonstrated that miRNA expression profiling can classify GEM models according to luminal or basal subtypes and that relatively few miRNAs are expressed in a model-specific manner despite different initiating oncogenic drivers used in the design of the models. Although these results strongly suggest that the miRNA expression patterns primarily reflect the state of tumor cell differentiation (luminal versus basal), more subtle distinctions in miRNA expression can be identified in the different models.

The differential expression of miRNAs among the eight murine models resulted in their segregation into several clusters. One major cluster included the *p53*^-/- ^transplant and C3(1)/SV40 *T/t*-antigen GEM models. These two models both develop mammary tumors with basal features, suggesting that the associated miRNAs reflect the phenotype of the basal tumor lineage. Both of these model systems share mechanistic similarities through the loss of *p53 *function. SV40 Tag sequesters *p53 *by forming a Tag-*p53 *complex, thus inactivating *p53 *tumor suppressor function leading to abnormalities in cell cycle regulation, apoptotic response, genome instability and tumorigenesis [37]. These findings suggest that a common mechanism of miRNA deregulation may be involved in *p53*-mediated tumorigenesis. Although clustered within the basal group of tumors, the *BRCA1*^-/-^*p53*^+/- ^model forms an independent cluster, which may indicate that these tumors express distinct molecular features as has been suggested previously [13].

Another major cluster of tumors includes four of the MMTV-promoter driven GEM models - MMTV-*H-Ras*, MMTV-*PymT*, MMTV-*Her2/neu*, and MMTV-*Wnt1*- that develop mammary tumors with more luminal features. Interestingly, there was some overlap between the miRNA expression patterns between these mouse mammary tumors with luminal features and the normal mammary gland, further suggesting that the miRNA expression pattern of these tumors is related to a luminal phenotype. This is consistent with a previous report that a cluster of luminal breast cancer miRNAs may be involved in the control of normal mammary gland development and become deregulated in breast cancer [38]. Nevertheless, our findings that the MMTV-driven tumors cluster with normal mammary glands also suggest that the MMTV LTR may target expression to a mammary cell lineage with luminal characteristics. Mammary epithelial cells in the pregnant mammary gland are in a state of increased proliferation and differentiation. This may also contribute to the clustering of the normal pregnant glands with the MMTV promoter-driven tumors.

We identified a signature of 122 miRNAs that are associated with the basal-like mammary tumors, and a signature of 73 miRNAs associated with the luminal-type mammary tumors. Blenkiron *et al*. [7] reported 38 miRNAs that are differentially expressed among human basal-like, HER2+, luminal A, luminal B or normal-like tumor subtypes, and these miRNAs have been shown to be involved in mammary gland development [38]. Importantly, we find that several of these miRNAs are consistent with our findings in the GEM models. Three miRNAs associated with human basal-type tumors (miR-135b, miR-505 and miR-155), and seven miRNAs associated with human luminal type tumors (let-7a, let-7f, miR-100, miR-130a, miR-152, miR-214 and miR-29b) are similarly expressed in mouse basal-like and luminal-type tumors, respectively. This suggests that the expression of these miRNAs may be evolutionarily conserved during mammary tumor differentiation. Therefore, the mouse models described may prove useful for understanding tumor lineage specification and how miRNAs play a role in this process.

Many of the miRNAs that we have identified to be associated with luminal type GEM tumors have been shown to be expressed at various stages of normal murine mammary gland development. Avril-Sassen *et al*. [38] identified seven miRNA clusters with distinct patterns of expression during mouse mammary gland development. Many of the miRNAs we have identified as being primarily expressed in luminal type GEM mammary tumors are found in two of these miRNA clusters. miR-193, -30b, -30c, -26a, and -26b are highly expressed during early development, gestation and late involution; miR-141, -200a, -148a, and -146b are highly expressed during gestation, lactation, and early and late involution. These results suggest that the various mouse luminal-type tumors induced by the MMTV LTR-targeted expression of oncogenes maintain specific luminal miRNA expression patterns, although the cells have become tumorigenic. Interestingly, the mRNA expression patterns of several oncogene-induced GEM tumor models driven by the MMTV LTR also cluster together despite utilizing oncogenes that function in different oncogenic pathways. This suggests that the MMTV LTR in these models may be targeting a particular mammary luminal epithelial cellular compartment at a specific stage of differentiation, resulting in tumors that share many similarities in miRNA and mRNA expression.

Several of the miRNAs that we have identified as being specific for the luminal-type GEM tumors (miR-141, -200a and -200b) have been shown to repress an EMT [39-41]. miR-141 inhibits EMT in part through targeting of transforming growth factor-β2. miR-200a has been shown to repress EMT through targeting of β-catenin. The miR-200 family has also been shown to target SIP1 and ZEB1, which are mediators of EMT. Thus, expression of miR-141, -200a and -200b in luminal tumors is in keeping with maintenance of the luminal phenotype.

Comparison of miRNA expression of normal mammary epithelium from glands harvested at day 17.5 of gestation to the GEM tumors identified several miRNAs that were primarily expressed only in the normal epithelium. Interestingly, we identified five miRNAs - miR-10b, -148a, -150, -199a and -486 - that are down-regulated in all of the mammary tumors compared to normal mammary gland tissue irrespective of the initiating genetic lesion. Four of these miRNAs - miR-10b, -148a, -150, -199a - have been implicated in mouse mammary gland development [38]. One of these, miR-10b, has been shown to be down-regulated in human breast carcinoma compared to normal breast tissue. miR-10b, which targets *HOXD10*, was additionally shown to be down-regulated in all the breast carcinomas from metastasis-free patients [42]. miR-199a functions as an onco-suppressor targeting the oncogene *Met*, therefore impairing Met-mediated invasive growth of cells [43]. miR-150 has been shown to negatively regulate the expression of the *Myb *oncogene [44]. These findings suggest that the loss of some or all of these miRNAs may be important for tumor development. miR-486, also expressed in normal epithelium, has been shown to be down-regulated in mammary cancer. Together, these data suggest that these miRNAs might function as tumor suppressors or regulate cellular differentiation and become deregulated during mammary tumor development.

Interestingly, although we identified many miRNAs whose expression was observed in basal type tumors, few of these miRNAs have been previously characterized. Thus, these basal GEM mammary tumor models may offer an important opportunity to delineate the functions of these less well studied basal-associated miRNAs.

Relatively few miRNAs were identified as being specifically expressed in particular GEM models. miR-22 was found to be primarily expressed in MMTV-*Wnt1 *tumors. miR-22 has previously been shown to be over-expressed in progenitor cells [45]. This would be in keeping with earlier studies that have suggested that MMTV-*Wnt1 *tumors are enriched for cells with stem cell characteristics [46-49]. Three miRNAs were found to be highly expressed in the *c-Myc *model, including miR-494, miR-699 and miR-685. Among them, miR-494 is highly associated with the luminal-type of mammary tumors, suggesting a potential role for miR-494 in *c-Myc*-mediated oncogenic signaling and in mammary tumor differentiation. miR-494 is highly expressed in human retinoblastoma [50]. It also negatively regulates *PTEN *gene expression at the translational level in human bronchial epithelial cells induced by anti-benzo(a)pyrene-trans-7,8-dihydrodiol-9,10-epoxide (anti-BPDE) and functions as a micro-oncogene in carcinogenesis [51].

Furthermore, by using an integrated miRNA and mRNA gene expression analysis, we demonstrated *in vivo *that the expression of miRNAs can be associated with the inverse expression of a subset of predicted target mRNAs in mammary gland tumors, leading to a more focused set of miRNAs to functionally validate. Since computational prediction of miRNA targets is inconsistent across different algorithms and usually identifies hundreds of potential targets, our approach of identifying an inverse correlation between miRNA and mRNA significantly reduces the number of potential candidates. However, it must be remembered that this analysis does not consider inhibition of protein translation by miRNA, which has been considered the primary mode of action of miRNAs. Therefore, additional miRNA targets need to be considered at the protein level. However, whether miRNA works primarily through inhibition of translation or transcription remains controversial [52].

Real-time RT-PCR demonstrated that the expression of *Birc4 *was reduced in mammary tumor epithelial cells that over-expressed miR-494. However, further analyses will confirm that miR-494 targets the putative mRNA sequence in the 3' UTR of *Birc4*. miR-412 was the only miRNA associated specifically with the C3(1)Tag model, and is also highly associated with the basal-like mammary tumors. Real-time RT-PCR demonstrated that overexpression of miR-412 reduces expression of *Bmpr1a*. Identification of the mRNA target in the 3' UTR of *Bmpr1 *will validate this finding. Bmpr1a is a type 1A bone morphogenetic protein receptor, but its functional role in breast cancer has not been defined. Decreased expression of Bmpr1b predicts poor prognosis in breast cancer patients and leads to increased cell proliferation of breast cancer cells *in vitro*, suggesting the tumor suppressor role of the Bmpr family in breast cancer carcinogenesis [53]. Therefore, inhibition of *Bmpr1a *expression by miR-412 could be involved in tumor initiation or progression of the C3(1)Tag and basal models.

## Conclusions

miRNA expression patterns in GEM models provides novel new insights into the associations between miRNA expression, mammary tumor subtypes and oncogenic drivers. Ongoing functional studies will determine the biologic roles that these miRNAs play in mammary epithelial differentiation, tumor suppression and oncogenesis.

## Materials and methods

### Animals

All the transgenic mice studied were of the FVB strain background except that *p53*^-/- ^and *BRCA1*^-/-^*p53*^+/- ^knockout mice were of the Balb/C and 129B6/FVB background, respectively. All the mice were housed and cared for in accordance with National Institutes of Health guidelines under an approved animal protocol. Tumors were harvested at the 0.5 to 1 cm stage, fixed in 4% (w/v) paraformaldehyde for histology, and the remainder snap frozen in liquid nitrogen. Tumors from four to seven individual mice were analyzed for each mouse model. Mammary glands from normal pregnant female mice at 17.5 days of pregnancy were also collected from the FVB, Balb/C and 129B6/FVB strains.

### miRNA cloning and lentivirus packaging

miR-412 and miR-494 were PCR amplified from C57/B6J mouse genomic DNA. The PCR fragment containing the miRNA stem loop sequence plus both the upstream and downstream flanking genomic sequence was then cloned into the plemiR lentiviral vector (Openbiosystems, Huntsville, AL, USA). The primers used were: miR-412, 5'- TCG ACT CGA GCA ACT TTG CAT CTG GAG GAC -3' and 3'- TCG AAC GCG TTG AGC GTT GAT ACT G AG AAA AGA T -5'; miR-494, 5'-TCG ACT CGA GCA CAG GGG TTT TGG TTG C -3' and 3'- TCG AAC GCG TGG GCT GAG TCC TGA TGC -5'.

Lentivirus plemiR-miR412 and plemiR-miR494 were prepared in 293T cells using the third-generation lentivirus packaging system.

### Cells and lentivirus infection

M6 and DB7 are mouse mammary tumor epithelial cell lines: M6 cells are derivative of primary tumors developed from C3(1)/SV40 *T/t*-antigen transgenic mice; DB7 cells are derivative of primary tumors developed from MMTV-*PymT *transgenic mice. Cells were transduced with plemiR lentivirus expressing miR-494, miR-412, or plemiR_scramble lentivirus as control. Following transduction, cells were grown in culture under puromycin (1 μg/ml) selection, and subsequently were sorted for RFP expression by FACS.

### RNA extraction

The total RNA containing the miRNA species were extracted from tumor samples using a *mir*Vana miRNA Isolation kit (Ambion, Austin, TX, USA). The RNA quality and yields were analyzed using Agilent Bioanalyzer and Nanodrop. Each RNA sample was then divided into two aliquots that were applied either for the miRNA microarray or the Affymetrix mRNA microarray.

### miRNA microarray

The miRNA microarray chip (LMT_miRNA_v2 microarray) was designed using the Sanger miR9.0 database [54] and manufactured by Agilent Technologies as custom-synthesized 8 × 15k microarrays. The array contains 1,667 unique mature miRNA sequences across all species, among them 334 unique miRNAs for mouse. Each mature miRNA is represented by + and - (reverse complement) strand sequences, and each with four replicate probes. In addition, the array contains both positive and negative controls, and other controls such as probes to *Actin, GAPDH, HSP70*, and LINE elements. The mature miRNA sequences were incorporated into 60-mer long oligonucleotide probes with a linker sequence on the 3' end to remove the miRNA sequences away from the glass slide surface. The linker sequence was a proprietary sequence from Agilent that has minimal homology to any sequence in the GenBank.

Total RNA (1 μg) containing the miRNAs was labeled using the miRCURY ™ LNA microRNA Array Labeling kit (Exiqon, Woburn, MA, USA). The 3' end of the total RNA was enzymatically labeled with the Hy3 and/or Hy5 fluorescent dye (Exiqon) by incubating with T4 RNA ligase at 0°C for 1 hour followed by an enzyme inactivation step of 65°C for 15 minutes. The labeled RNA was subsequently used for hybridization onto the microarrays without the need for column purification.

The fluorescence-labeled miRNAs were incubated with a 2 × hybridization buffer and 10 × blocking buffer (both from Agilent). The samples were subsequently heated to 99°C for 3 minutes, snap-cooled on ice, and centrifuged for 5 minutes before being added onto the microarray printed on glass slides and hybridized for 16 hours at 47°C inside the Agilent hybridization rotating oven. After the 16-hour incubation overnight, the glass slides containing the microarrays were washed with Agilent wash buffers 1 (room temperature) and 2 (at 37°C) and then dried with the Agilent stabilization and drying solution. The washed and dried slides were scanned using the Agilent scanner. The Feature Extraction program was used to extract the spot intensities.

### Gene expression microarray

Total RNA (1 μg) was reverse transcribed with T7-oligo(dT) primer and labeled with biotin using Affymetrix One Cycle Target Labeling kit following the manufacturer's protocol. RNA was then labeled and hybridized to the mouse genome 430A 2.0 GeneChip (Affymetrix) and scanned on an Affymetrix GeneChip scanner 3000. Data were collected using Affymetrix GCOS software.

### miRNA microarray data analysis

#### miRNA gene expression data normalization

The gProcessSignal values of probes designed for mouse miRNAs were feature extracted using the GE2 protocol (Agilent) with exclusion of internal control probes, non-mouse probes, and all negative strand probes. A global median normalization procedure was applied to the gProcessSignal values of the selected probes across all arrays. The normalized data were further filtered using MAS5 detection calls ('P' (Present), 'M' (Marginal), or 'A' (Absent)) to eliminate probes with 'P' or 'M' in less than three samples in the entire dataset.

#### Unsupervised hierarchical clustering

Heatmaps and hierarchical clustering were performed using TM4 MeV from TIGR [55] or the Partek Genomic Suite [56] using z-scores transformed from the original normalized values.

#### Identification of basal-luminal specific miRNAs

For comparison of basal and luminal model samples, differential miRNA were derived using SAM (significance analysis of microarray) [57] under cutoff *P *≤ 0.01 and FDR ≤0%. The normal mammary samples were then integrated into the heatmap for comparison. After selection of basal-luminal differentially expressed miRNAs, the transformed z-scores of these selected miRNAs were visualized and displayed in the form of heatmaps using TM4 MeV from TIGR [55] or the Partek Genomic Suite [56].

#### Identification of mammary cancer model-specific miRNAs

Model-specific miRNA signatures were derived from in-house z-score-based methods. Briefly, all probe signal intensity values were transformed into z-scores. The mouse model-specific expression of an miRNA was defined as the miRNAs with z-scores > 0.75 within the particular model, and with the median z-score of the particular model higher than the third highest ranked z-scores of pooled samples of all other models. *P*-values and FDRs were derived from sample-labeling permutation or directly based on the Benjamini and Hochberg method (FDR-BH) [58]. *t*-Test *P*-values and related FDRs were also reported for the two-class comparisons of the particular model versus other models. The *P*-values for feature selection were generally less than 0.001 and the FDR by permutation test less than or close to 1% and FDR-BH < 5%. We observed that these methods, in fact, performed better than an ANOVA-based approach, probably due to the fact that the sample size is limited for each model and our methods are more stringent and conservative. Our method resulted in a more conservative model-specific pattern. After selection of model-specific miRNA signatures, the transformed z-scores of these selected miRNAs were visualized and displayed in the form of heatmaps using TM4 MeV from TIGR [55] or the Partek Genomic Suite [56].

### miRNA-mRNA negative correlation and enrichment analysis

mRNA array data were normalized using GC-RMA of the Partek Genomic Suite [56]. The normalized data were further filtered using MAS5 detection calls for probes designated as 'P' (present) or 'M' (Marginal) in less than three samples from all of the samples analyzed. Basal-luminal differential miRNAs and model-specific miRNA signatures were derived as described above. Analysis to identify negative correlations between miRNA and mRNA expression was done using an in-house R script. Briefly, normalized miRNA and mRNA data were sample-matched for all samples with both miRNA and mRNA array data. Then for each miRNA (either differential miRNA between basal and luminal or model-specific signature miRNA), Pearson correlation coefficients were computed for all mRNAs. The predicted target mRNAs of the particular miRNA were selected from the TargetScan database [54], and the Pearson correlation coefficients between the particular miRNA and its predicted target mRNAs were computed as well. For each miRNA, a 2 × 2 contingency table was created for all mRNAs (whether a mRNA has negative correlation with the intended miRNA or not versus whether it is a predicted target of the intended miRNA or not), which was used to assess the enrichment level of the negative correlated mRNAs (correlation < 0 and *P*-value of correlation ≤0.001) within predicted targets of the intended miRNA using Fisher's exact test. If the *P*-value of Fisher's exact test is less than 0.05, the miRNA is considered to have a significant number of mRNA targets with negative correlation with it and it was selected as a significant miRNA in this screening procedure. Then for each significant miRNA, the distribution of correlation coefficients (cor) for both target mRNAs and all mRNAs was also plotted to confirm the significant left shift of the distribution curve of the target mRNAs towards the negative correlation side compared to the curve for all mRNAs. The shift of the distribution plots between the target mRNAs and all mRNAs indicates enrichment of the target mRNAs (Fisher *P *< 0.05).

### Double immunofluorescence assay

Paraffin-embedded sections (5 μm thick) were processed using sequential immunostaining for cytokeratin 14 (K14) and cytokeratin 18 (K18) using standard procedures. Briefly, slides were deparaffinized followed by antigen retrieval, and blocked with serum. Slides were then incubated overnight with rabbit α-cytokeratin 14 (1:20,000; PRB-155P, Covance, provided by Dr SH Yuspa, NIH) at 4°C, blocked with avidin/biotin (Vector Labs #SP-2001, Burlingame, CA, USA) followed by incubation with sheep α-cytokeratin 18 (1:800, #PH504, The Binding Site, San Diego, CA, USA) overnight at room temperature. Slides were then stained for 30 minutes with biotin-conjugated donkey α-rabbit (1:100; Abcam #AB6801, Cambridge, MA, USA) and rabbit α-sheep (1:100; Vector Labs #BA-6000) secondary antibody, followed by streptavidin-conjugated Alexa fluor-594 or -488 (1:100; Invitrogen #s S11227 and S11223, Carlsbad, CA, USA), respectively. Slides were also counter-stained with DAPI.

### Quantitative real-time RT-PCR for miRNAs

Taqman miRNA assays (Applied Biosystems, Carlsbad, CA, USA) were performed to measure the expression of miRNAs following the manufacturer's protocol. For miRNAs miR-30b, -412 and -505, SYBR-based miScript miRNA assays (Qiagen, Valencia, CA, USA) were performed to measure their expression following the manufacturer's protocol. The relative quantification of mature miRNA expression was normalized to the expression of endogenous mouse snoRNA-202.

### Quantitative real-time RT-PCR for gene expression

Total RNA was isolated as mentioned above. First-strand cDNA was synthesized using the SuperScript III First-Strand synthesis system (Invitrogen). Quantitative real-time RT-PCR was then performed using iQ SYBR Green supermix (Bio-Rad, Hercules, CA, USA) in triplicates (MyiQ single-color real-time PCR detection system, Bio-Rad). The relative quantification of gene expression was normalized to the expression of the endogenous gene *GAPDH*.

Primer sequences were: *GAPDH*, 5'- CAT GGC CTT CCG TGT TCC TA-3' and 3'- GCG GCA CGT CAG ATC CA -5'; Cycophilin, 5'-TGC TGG ACC AAA CAC AAA CG-3' and 3'-CCA TCC AGC CAT TCA GTC TTG-5'; *Bmpr1a*, 5'- AAC GCT TGC GGC CAA TC -3' and 3'- GAC ATT AGC TTC AAA ACT GCT CGA A -5'; *Bmi1*, Mm_Bmi1_1_SG, #QT00165298 (Qiagen); *Spry4*, Mm_Spry4_1_SG, #QT00263844 (Qiagen); *Birc4*, #VMPS-383 (http://RealTimePrimers.com); Foxo3a, #VMPS-28 (http://RealTimePrimers.com).

### GEO submission of microarray data

Data have been deposited with the Gene Expression Omnibus: miRNA gene expression raw data (before normalization) [GSE23978]; miRNA gene expression raw data of normal mammary gland tissues from different mouse genetic background [GSE23977]; mRNA gene expression raw data [GSE23938].

## Abbreviations

EMT: epithelial-to-mesenchymal transition; ER: estrogen receptor; FACS: fluorescence activated cell sorting; FDR: false discovery rate; GEM: genetically engineered mouse; LTR: long terminal repeat; miRNA: microRNA; MMTV: mouse mammary tumor virus; PCR: polymerase chain reaction; PyMT: polyoma middle T antigen; RFP: red fluorescence protein; RT-PCR: reverse transcription PCR;UTR: untranslated region.

## Competing interests

The authors declare that they have no competing interests.

## Authors' contributions

MZ contributed to the design and conception of the experiments, conducted molecular biology experiments, analyzed and interpreted data and drafted the manuscript. CHK performed the microarray experiments and helped with quality control and analysis. MY and RS normalized the data and performed all statistical analyses. CD and DM provided tumor tissue samples that they had characterized. JEG conceived of the project and participated in its design, helped to analyze and interpret the data and draft the manuscript. All authors have read and approved the manuscript for publication.

## Supplementary Material

Additional file 1 **Figure S1 - miRNA gene expression profile of normal mammary gland tissues from different mouse genetic backgrounds**. The miRNAs of the normal mammary glands are compared to those of the C3(1)/Tag mammary tumors as a control. Click here for file

Additional file 2 **Figure S2 - unsupervised hierarchical clustering of the 22 differentially expressed miRNA genes identified in **Additional file 1**over 41 mammary tumors derived from 8 genetically engineered mouse models and 5 normal mammary tissues**. The heatmap shows the expression of miRNAs at the probe level. Heatmap colors represent relative miRNA expression as indicated in the color key. Click here for file

Additional file 3 **Figure S3 - double-immunofluorescence staining of mouse samples for basal/myoepithelial and luminal cytokeratins**. Normal mammary gland and mammary tumors from the indicated mouse models are stained for cytokeratin 18 (K18; green) and cytokeratin 14 (K14; red). Click here for file

Additional file 4 **Figure S4 - correlation of miRNA microarray data with quantitative RT-PCR miRNA expression data**. Shown are the pairwise scatter plots for individual miRNAs. The y-axis of the plot shows the log2 intensity of the microarray data, whereas the x-axis shows the -delta cycle threshold (CT) value of the RT-PCR results. Each dot in the plot represents one sample from individual tumor models or normal mammary tissues. Person correlation coefficients (r) and *P*-values are calculated. Click here for file

Additional file 5 **miRNAs that are highly associated with basal- and luminal- mammary tumor subtypes**. Click here for file

Additional file 6 **miRNAs that are highly associated with individual genetically engineered mouse models and normal mammary tissues**. Click here for file

Additional file 7 **Genetically engineered mouse model-specific miRNAs and their potential mRNA targets**. Click here for file

Additional file 8 **Basal- or luminal-like miRNAs and their potential mRNA targets**. Click here for file

Additional file 9 **Figure S5 - analysis of the inverse relationship between transcript levels of miRNAs and their putative target mRNAs in mouse mammary tissues**. Global distribution of the Pearson correlation coefficients between mRNAs and **(a) **miR-10b, **(b) **miR-412 and **(c) **miR-494. The dotted curves show the distribution of the correlation coefficients for all mRNAs. The solid curves show the correlation coefficients for only those mRNAs that are predicted targets of miR-10b, miR-412 or miR-494. Click here for file

Additional file 10 **Figure S6 - Ingenuity Pathway Analysis™ of the potential target genes of miR-494**. Twelve of the mRNA target genes of miR-494 from Table 3 were input into Ingenuity (Ingenuity Systems, Inc.), and core analysis was then performed to retrieve the target genes' association with cancer and disease. Click here for file

Additional file 11 **Figure S7 - overexpression of miR-494 in M6 cells as determined by quantitative real-time RT-PCR**. M6 cells were transduced with plemiR lentivirus expressing miR-494. Control cells were M6 cells and M6 cells transduced with plemiR lentivirus vector. Following infection, cells were FACS sorted for RFP and RNA was extracted. Real-time RT-PCR was then performed to examine the expression of miR-494 in these cells. Click here for file

Additional file 12 **Figure S8 - overexpression of miR-494 in M6 cells does not alter expression of *Bmi1 *or *Ptpn12 *determined by quantitative real-time RT-PCR**. M6 cells were transduced with plemiR lentivirus expressing miR-494. Control cells were M6 cells, M6 cells transduced with plemiR lentivirus vector, and M6 cells transduced with lentivirus expressing scrambled miRNA. Following infection, cells were FACS sorted for RFP and RNA was extracted. Real-time RT-PCR was then performed to examine the expression of *Bmi1 *(top) and *Ptpn12 *(bottom) in these cells. *P*-value (*Bmi1*: M6_miR494 versus M6_scramble) = 0.06; *P*-value (*Ptpn12*: M6_miR494 versus M6_scramble) = 0.0502. Click here for file

Additional file 13 **Figure S9 - increased expression of *Foxo3a *and *Spry4 *by miR-412 in DB7 cells**. DB7 cells were transduced with plemiR lentivirus expressing miR-412. Control cells were DB7 cells, DB7 cells transduced with plemiR lentivirus vector, and DB7 cells transduced with lentivirus expressing scrambled miRNA. Following infection, cells were FACS sorted for RFP and RNA was extracted. Real-time RT-PCR was then performed to examine the expression of *Foxo3a *and *Spry4 *in these cells. *P*-value (*Foxo3a*: DB7_miR412 versus DB7_scramble) = 0.125; *P*-value (*Spry4*: DB7_miR412 versus DB7_scramble) = 2.75E-06. Click here for file
