# Supplementary material for: Integrated miRNA and mRNA expression profiling of mouse mammary tumor models identifies miRNA signatures associated with mammary tumor lineage
Source: Genome Biol. 2011 Aug 16;12(8):R77. doi: 10.1186/gb-2011-12-8-r77 (PMC3245617; doi:10.1186/gb-2011-12-8-r77)
Supplement: Additional file 1 — Figure S1 - miRNA gene expression profile of normal mammary gland tissues from different mouse genetic backgrounds. The miRNAs of the normal mammary glands are compared to those of the C3(1)/Tag mammary tumors as a control. [file gb-2011-12-8-r77-S1.PDF]

miRNA gene expression profiles of normal mammary gland tissues from different mouse genetic backgrounds.

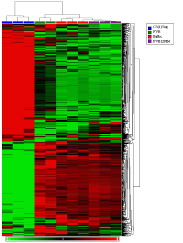

Additional File 1, Figure S1
