# Supplementary material for: Integrated miRNA and mRNA expression profiling of mouse mammary tumor models identifies miRNA signatures associated with mammary tumor lineage
Source: Genome Biol. 2011 Aug 16;12(8):R77. doi: 10.1186/gb-2011-12-8-r77 (PMC3245617; doi:10.1186/gb-2011-12-8-r77)

Additional Figure 2..
Unsupervised hierarchical clustering of the 22 differentially expressed miRNA genes identified in Supplemental Figure 1A over 41 mammary tumors derived from 8 GEM models and 5 normal mammary tissues.The heatmap shows the expression of miRNAs at the probe level. Heatmap colors represent relative miRNA expression as indicated in the color key.

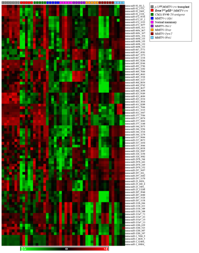

Supplement: Additional file 2 — Figure S2 - unsupervised hierarchical clustering of the 22 differentially expressed miRNA genes identified in Additional file 1over 41 mammary tumors derived from 8 genetically engineered mouse models and 5 normal mammary tissues. The heatmap shows the expression of miRNAs at the probe level. Heatmap colors represent relative miRNA expression as indicated in the color key. [file gb-2011-12-8-r77-S2.PDF]
