# Supplementary material for: Integrated miRNA and mRNA expression profiling of mouse mammary tumor models identifies miRNA signatures associated with mammary tumor lineage
Source: Genome Biol. 2011 Aug 16;12(8):R77. doi: 10.1186/gb-2011-12-8-r77 (PMC3245617; doi:10.1186/gb-2011-12-8-r77)
Supplement: Additional file 3 — Figure S3 - double-immunofluorescence staining of mouse samples for basal/myoepithelial and luminal cytokeratins. Normal mammary gland and mammary tumors from the indicated mouse models are stained for cytokeratin 18 (K18; green) and cytokeratin 14 (K14; red). [file gb-2011-12-8-r77-S3.PDF]

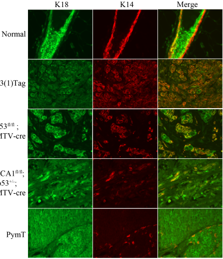

Additional File 3, Figure S3. Double-immunofluorescence staining of mouse samples for basal/myoepithelial and luminal cytokeratins. Normal mammary glands and mammary tumors derived from C3(1)/SV40 T/t-antigen, p53fl/fl ;MMTV-cre, BRCA1fl/fl;p53+/-;MMTV-cre, MMTV-PymT mice are stained for both cytokeratin 18 (K18) and 14 (K14, red).The staining shows K18 expression in luminal epithelial cells (green) and K14 expression in basal/myoepithelial cells (red).
