# Supplementary material for: Integrated miRNA and mRNA expression profiling of mouse mammary tumor models identifies miRNA signatures associated with mammary tumor lineage
Source: Genome Biol. 2011 Aug 16;12(8):R77. doi: 10.1186/gb-2011-12-8-r77 (PMC3245617; doi:10.1186/gb-2011-12-8-r77)
Supplement: Additional file 4 — Figure S4 - correlation of miRNA microarray data with quantitative RT-PCR miRNA expression data. Shown are the pairwise scatter plots for individual miRNAs. The y-axis of the plot shows the log2 intensity of the microarray data, whereas the x-axis shows the -delta cycle threshold (CT) value of the RT-PCR results. Each dot in the plot represents one sample from individual tumor models or normal mammary tissues. Person correlation coefficients (r) and P-values are calculated. [file gb-2011-12-8-r77-S4.PDF]

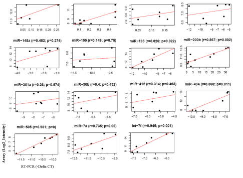

Additional File 4, Figure S4. Correlation of miRNA microarray data with quantitative real-time RT-PCR miRNA expression data. Shown are the pairwise scatter plots for individual miRNAs. The Y-axis of the plot shows the log2 intensity of the microarray data, whereas the X-axis shows the –Delta CT value of the RT-PCR results. Each dot in the plot represents one sample from individual tumor models or normal mammary tissues.Pearson correlation coefficients (r) and P-value are calculated.
