# Supplementary material for: Integrated miRNA and mRNA expression profiling of mouse mammary tumor models identifies miRNA signatures associated with mammary tumor lineage
Source: Genome Biol. 2011 Aug 16;12(8):R77. doi: 10.1186/gb-2011-12-8-r77 (PMC3245617; doi:10.1186/gb-2011-12-8-r77)
Supplement: Additional file 9 — Figure S5 - analysis of the inverse relationship between transcript levels of miRNAs and their putative target mRNAs in mouse mammary tissues. Global distribution of the Pearson correlation coefficients between mRNAs and (a) miR-10b, (b) miR-412 and (c) miR-494. The dotted curves show the distribution of the correlation coefficients for all mRNAs. The solid curves show the correlation coefficients for only those mRNAs that are predicted targets of miR-10b, miR-412 or miR-494. [file gb-2011-12-8-r77-S9.PDF]

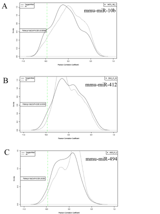

Additional File 9, Figure S5. Analysis of the inverse relationship between transcript levels of miRNAs and their putative target mRNAs in mouse mammary tissues. Global distribution of the Pearson correlation coefficients between mRNAs and (a) miR-10b; (b) miR-412 and (c) miR-494. The dotted curves show the distribution of the correlation coefficients for all mRNAs. The solid curves show the correlation coefficients for only those mRNAs that are predicted target of miR-10b, miR-412 or miR-494.
