# Supplementary material for: Integrated miRNA and mRNA expression profiling of mouse mammary tumor models identifies miRNA signatures associated with mammary tumor lineage
Source: Genome Biol. 2011 Aug 16;12(8):R77. doi: 10.1186/gb-2011-12-8-r77 (PMC3245617; doi:10.1186/gb-2011-12-8-r77)
Supplement: Additional file 10 — Figure S6 - Ingenuity Pathway Analysis™ of the potential target genes of miR-494. Twelve of the mRNA target genes of miR-494 from Table 3 were input into Ingenuity (Ingenuity Systems, Inc.), and core analysis was then performed to retrieve the target genes' association with cancer and disease. [file gb-2011-12-8-r77-S10.PDF]

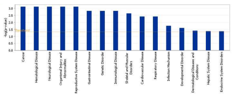

© 2000-2009 Ingenuity Systems, Inc. All rights reserved.

Additional File 10, Figure S6. Ingenuity analysis of the potential target genes of miR-494. Twelve of the mRNA target genes of miR-494 from Table 3a were input into Ingenuity (Ingenuity Systems, Inc.), and core analysis was then performed to retrieve the target genes' association with cancer and disease.
