# Supplementary figures and images for: Integrated miRNA and mRNA expression profiling of mouse mammary tumor models identifies miRNA signatures associated with mammary tumor lineage
Source: Genome Biol. 2011 Aug 16;12(8):R77. doi: 10.1186/gb-2011-12-8-r77 (PMC3245617; doi:10.1186/gb-2011-12-8-r77)

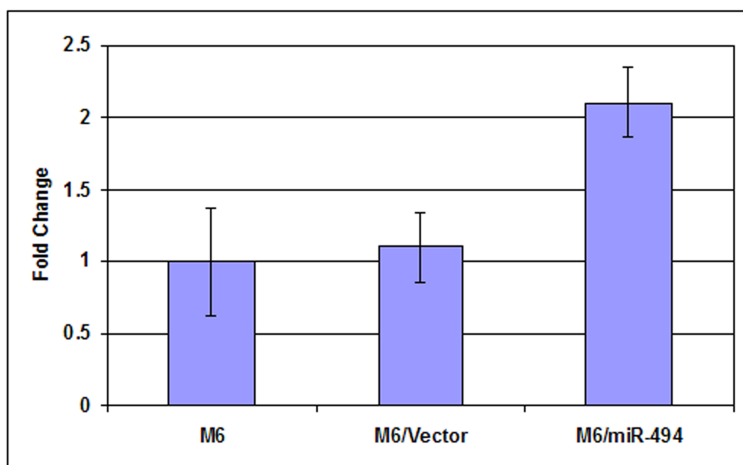

Additional File 11, Figure S7

Supplement: Additional file 11 — Figure S7 - overexpression of miR-494 in M6 cells as determined by quantitative real-time RT-PCR. M6 cells were transduced with plemiR lentivirus expressing miR-494. Control cells were M6 cells and M6 cells transduced with plemiR lentivirus vector. Following infection, cells were FACS sorted for RFP and RNA was extracted. Real-time RT-PCR was then performed to examine the expression of miR-494 in these cells. [file gb-2011-12-8-r77-S11.PDF]

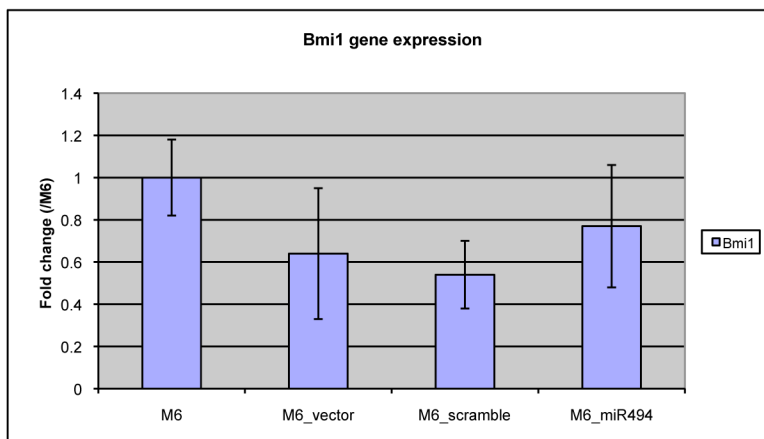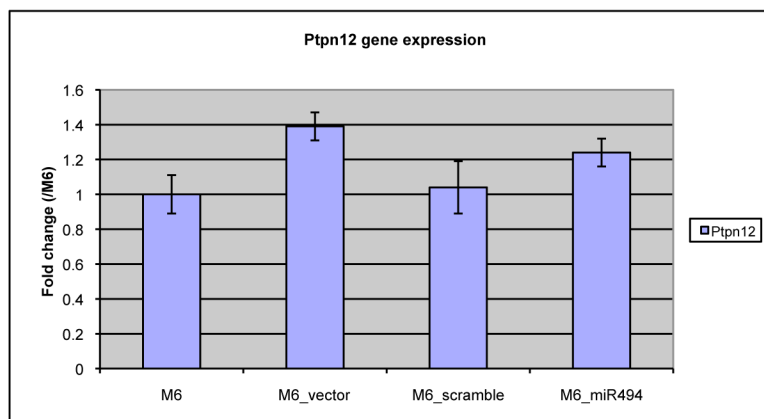

Additional File 12, Figure S8.

Supplement: Additional file 12 — Figure S8 - overexpression of miR-494 in M6 cells does not alter expression of Bmi1 or Ptpn12 determined by quantitative real-time RT-PCR. M6 cells were transduced with plemiR lentivirus expressing miR-494. Control cells were M6 cells, M6 cells transduced with plemiR lentivirus vector, and M6 cells transduced with lentivirus expressing scrambled miRNA. Following infection, cells were FACS sorted for RFP and RNA was extracted. Real-time RT-PCR was then performed to examine the expression of Bmi1 (top) and Ptpn12 (bottom) in these cells. P-value (Bmi1: M6_miR494 versus M6_scramble) = 0.06; P-value (Ptpn12: M6_miR494 versus M6_scramble) = 0.0502. [file gb-2011-12-8-r77-S12.PDF]

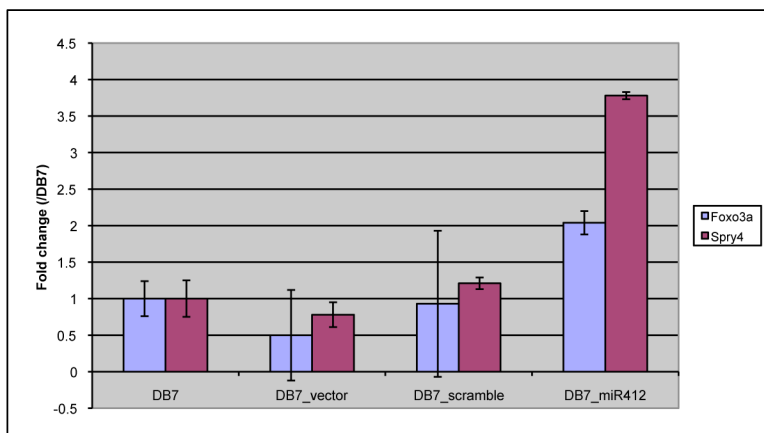

Additional File 13, Figure S9.

Supplement: Additional file 13 — Figure S9 - increased expression of Foxo3a and Spry4 by miR-412 in DB7 cells. DB7 cells were transduced with plemiR lentivirus expressing miR-412. Control cells were DB7 cells, DB7 cells transduced with plemiR lentivirus vector, and DB7 cells transduced with lentivirus expressing scrambled miRNA. Following infection, cells were FACS sorted for RFP and RNA was extracted. Real-time RT-PCR was then performed to examine the expression of Foxo3a and Spry4 in these cells. P-value (Foxo3a: DB7_miR412 versus DB7_scramble) = 0.125; P-value (Spry4: DB7_miR412 versus DB7_scramble) = 2.75E-06. [file gb-2011-12-8-r77-S13.PDF]
